# Editorial: Artificial intelligence applications in chronic ocular diseases

**DOI:** 10.3389/fcell.2023.1295850

**Published:** 2023-12-07

**Authors:** Yanwu Xu, Weihua Yang

**Affiliations:** ^1^ School of Future Technology, South China University of Technology, Guangzhou, Guangdong Province, China; ^2^ Pazhou Lab, Guangzhou, Guangdong Province, China; ^3^ Shenzhen Eye Institute, Shenzhen Eye Hospital, Jinan University, Shenzhen, Guangdong Province, China

**Keywords:** artificial intelligence, deep learning, machine learning, chronic ocular diseases, diagnosis, ocular structures analysis

## Introduction

Chronic ocular diseases are eye health conditions that develop slowly over an extended period, typically without showing noticeable symptoms in the short term. These conditions can progressively affect vision and overall ocular health, potentially causing significant disruptions to a patient’s life quality if not promptly diagnosed and treated. Common chronic ocular diseases include glaucoma (Morgan and Drance, 1975), cataracts, dry eye syndrome (Schaumberg et al., 2002), diabetic retinopathy, age-related macular degeneration, myopia (Singh et al., 2022), and hyperopia (Sharafeldin et al., 2018). It is essential for individuals with chronic ocular diseases to receive regular eye examinations and appropriate medical care to manage and mitigate the long-term effects of these conditions on their eye health and vision.

Artificial intelligence (AI) is a cutting-edge technology in computer science that aims to enable computers to learn, reason, and make decisions like humans. AI technology has already achieved great success in many fields, including automatic driving, financial forecasting, natural language processing applications, and medical diagnostics. Among the field of medical diagnostics, AI technology has significantly contributed to clinical research in various areas. These include AI-assisted diagnosis of intracranial tumors from magnetic resonance (MR) imaging scans (Anaraki et al., 2019), AI-supported analysis of vascular stenosis in coronary computed tomography (CT) imaging (Han et al., 2020), AI-assisted evaluation of head and neck CT angiography images (Fu et al., 2023), AI-powered diagnosis of fundus lesions from fundus imaging (Li et al., 2022), and AI-driven analysis of pneumonia in CT imaging (Chassagnon et al., 2021). Especially in eye imaging research, AI can be utilized to conduct studies in various areas. This includes retinal disease screening based on fundus color photographs (Orlando et al., 2020; Li et al., 2022), cataract grading based on AS-OCT images (Zhang et al., 2022), and glaucoma grading based on multi-modal ophthalmic examination images (Wu et al., 2023), such as fundus color photos and OCT images. AI can also be employed for the segmentation of ocular structures or lesions in different modal eye examination images, including optic cup and disc segmentation (Fu et al., 2018), retinal blood vessel segmentation (Hu et al., 2022), lesion segmentation (Fang et al., 2022) in fundus color images, and segmentation of ocular structural layers and lesions in OCT images (Li et al., 2021; Farshad et al., 2022).

Moreover, the application of AI technology in the management and treatment of chronic ocular diseases also holds significant importance. It can offer support and improvements across various aspects, including early diagnosis and prediction, disease progression monitoring, personalized treatment, etiological analysis and research, precise surgical assistance, and patient health management (Yang et al., 2023). AI techniques will be employed for intelligent image analysis, disease classification and diagnosis, surgical assistance, and etiological research.

**FIGURE 1:**
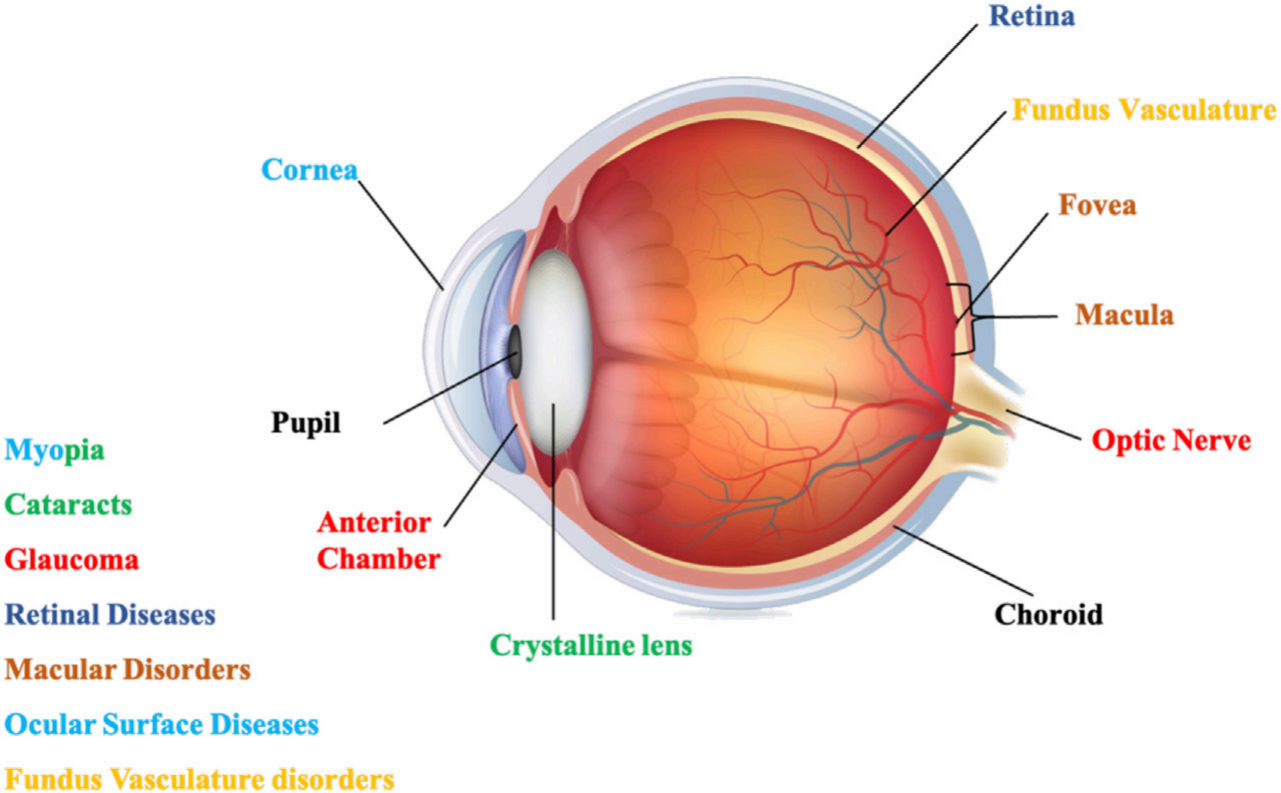
The structure of the eyeball, and chronic ocular diseases those can be caused by degeneration in different structures.

Hence, a Research Topic dedicated to the research on the application of AI in chronic ocular diseases has been initiated. During this Research Topic, we received a total of 53 submissions. These submissions underwent rigorous evaluation, resulting in the inclusion of 30 selected papers. The overall download count reached more than 7,600, with a combined total of 53k views and downloads. We have categorized the studies in this Research Topic according to the corresponding types of chronic diseases based on the structure of the eyeball from front to back, as illustrated in Figure 1; Table 1.

**TABLE 1:** Summary of the papers in the Research Topic of Artificial Intelligence Applications in Chronic Ocular Diseases.

Disease	Ocular structure	Paper	Main findings
Ocular Surface and orbital Diseases	Eye orbit and eyelids	Bao et al.	AI methods can automatically identify and quantify orbital anatomical structures
		Zhang et al.	
		Diao et al.	AI methods can provide applications in the clinical diagnosis, activity, severity grading, and treatment outcome prediction
		Wen et al.	
	Meibomian gland	Deng et al.	Using U-Net or its variants for the segmentation of the meibomian gland, followed by morphological parameter measurement, and subsequently applying the measured parameters for medical statistics on different diseases
		Yu et al.	
		Li et al.	
		Huang et al.	
	Cornea	Lin et al.	Using AI method to discuss the effects of cataract surgery and corneal incisions on corneal astigmatism
		Cheng et al.	Using deep learning method to measure the deviation of the pupil center during corneal refractive surgery
		Ji et al.	AI methods can automatically diagnosis ocular surface diseases
		Zhang et al.	
Myopia	Cornea	Wang et al.	Using AI methods for myopia risk prediction, diagnosis, screening, etc., and discussing the demonstrative role of AI methods in education
	Crystalline lens	Zhang et al.	
		Bai et al.	
	Choroid layer	Wu et al.	Designing a new boundary-enhanced encoder-decoder architecture for choroid layer segmentation
		Wu et al.	Employing AI methods to investigate the impact of drugs on the eye structure of myopia patients
Glaucoma	Anterior chamber	Zhang et al.	Introducing various diagnostic models for glaucoma based on different examine images. (Random forest models and VGG networks based on visual field test results; ResNet classifier based on fundus images; ResNet classifier based on OCT)
	Optic nerve		
Cataracts	Crystalline lens	Xie et al.	Developing a deep learning method for screening visually impaired cataract cases using fundus images
Retinal Diseases	Retina	Bai et al.	Using AI methods to screen and analyze various retinal diseases, especially multiple macular-related diseases, and also employing AI methods to analyze the biological significance of cytokines in the retina
	Macula	Song et al.	
	Fovea	Feng et al.	
Fundus Vasculature Disorders	Fundus vasculature	Ji et al.	Applying AI methods in the diagnosis and grading of retinal vascular diseases
		Shi et al.	Using AI methods to segment blood vessels in different modal images, measure related parameters, and then analyze changes in microvascular parameters before and after disease onset or surgical intervention using medical statistics
		Deng et al.	
		Zhang et al.	
		Shen et al.	
		Tang et al.	
		Su et al.	Proposing an attention-guided cascade network for retinal vessel segmentation
		Zhang et al.	Using AI method eliminating eyelash artifacts from ultra-wide-field fundus images, aiming to improve the visibility of retinal vasculature and enhance the quality of images

The ocular surface and orbital diseases involve structural lesions of the orbit, eyelids, cornea, and so forth. The orbit is the bony cavity within the skull that houses the eyeball, primarily serving to protect the eye. The eyelids are movable folds of skin that cover the eyeball, capable of opening and closing to protect the eye from external harm, regulate the entry of light, and secrete tears. The cornea is the transparent tissue on the front surface of the eye, allowing light to enter the eye. Myopia-related diseases may involve lesions on the cornea and the crystalline lens. The crystalline lens is a transparent biconvex structure within the eye, responsible for adjusting focal length. Glaucoma is related to structural changes in the anterior chamber and optic nerve pathology. The anterior chamber is a fluid-filled area located in the front part of the eye, between the iris and the cornea, responsible for maintaining the shape of the eye, supplying oxygen and nutrients, and regulating eye pressure. Cataract diseases exhibit significant abnormalities in the lens. Diseases of the retina involve lesions in areas such as the retina, macula, central fovea, and so forth. The primary function of retina is to perceive light, process visual information, and transmit this information to the brain. The macula is a critical area for vision, especially in detail resolution, color perception, and direct gaze. The central fovea is the most sensitive area of the retina, allowing us to achieve the highest resolution central vision. Fundus vasculature disorders are conditions caused by alterations in the retinal blood vessels. The primary function of retinal blood vessels is to supply the ocular tissues with the oxygen and nutrients.

## Ocular surface and orbital diseases

Ocular surface and orbital diseases are a group of eye conditions that impact the structure and function of the eye’s surface. Articles related to this topic in the Research Topic involve the studies of various aspects, including eye orbit, eyelids, meibomian glands, and cornea, as well as related diseases.

### Eye orbit and eyelids

Bao et al. and Diao et al. primarily focus on the orbital and eyelid images collected during clinical diagnostics for **Thyroid-Associated Ophthalmopathy (TAO)**. Bao et al. mainly discuss the application of AI technology in the analysis of orbital CT/MR images. For instance, AI methods can automatically identify and quantify orbital anatomical structures such as bone structures, fat, and abscesses using image segmentation techniques. Additionally, they summarize the applications of AI in TAO. Similarly, Diao et al. conduct research on the application of AI technology in the diagnosis dimension concerning TAO. They provide an overview of recent AI applications in the clinical diagnosis, activity, severity grading, and treatment outcome prediction. The study also discusses current challenges and future prospects in the application of AI in the treatment of TAO. Wen et al. study the difference of dynamic low-frequency amplitude between patients with TAO and normal people based on resting brain functional MR images (fMRI), laying a foundation for subsequent automatic diagnosis of thyroid-associated eye disease based on dynamic low-frequency amplitude. Based on eye orbit CT images, Zhang et al. focus on measuring eyeball protrusion by calculating eyeball protrusion distance using segmentation models in artificial intelligence.

### Meibomian gland

Four articles focus on meibomian gland analysis, all involving segmentation of the meibomian glands and subsequently performing different downstream tasks. Deng et al. segment the meibomian gland area to analyze the meibomian gland conditions in patients with meibomian gland dysfunction. Yu et al. conduct structural segmentation and morphological analysis of the meibomian glands to discuss the differences in meibomian gland morphology between patients with ophthalmic herpes zoster and a normal control group. Similarly, Li et al. analyze glandular model parameters, automatically calculating the glandular deformation coefficient to explore the impact of orthokeratology lenses (OK lenses) on tear film, meibomian glands, and myopia control in monocular myopic children. Huang et al. use a deep learning model to analyze images, measuring meibomian gland area, density, quantity, height, width, and curvature, and use a deep learning system to analyze the effects of age, gender, and behavior on meibomian gland morphology.

### Cornea

Among the intelligent analysis of the corneal structure, Lin et al. discuss the effects of cataract surgery and corneal incisions on corneal astigmatism. Cheng et al. mainly use deep learning technology to measure the deviation of the pupil center during corneal refractive surgery. The application, limitations, and challenges of AI in the diagnosis of ocular surface diseases (such as keratitis, keratoconus, dry eye, pterygium, and other ocular surface diseases), as well as the prospects of future applications are summarized by Ji et al. and Zhang et al.

## Myopia

The papers on this topic cover various aspects of myopia, including high myopia, pathological myopia, strabismus, amblyopia, and the effects of drugs on myopia. Specifically, Wang et al. provide a comprehensive review of the advancements in the application of different AI models and algorithms in optometry (for issues such as myopia, strabismus, amblyopia, keratoconus, and artificial lenses) and discussed the associated limitations and challenges in this field. Zhang et al. conduct a review and elaborated on the technical details of AI methods in myopia risk prediction, screening, diagnosis, pathogenesis, and treatment. Bai et al.discuss the application of AI-based myopia automatic recognition systems in the training of resident physicians, validating the role of artificial intelligence in medical education. Wu et al. employ AI technology to investigate the impact of drugs on the eye structure of myopia patients, particularly on choroidal thickness and retinal thickness. Additionally, Wu et al. conduct research on choroid layer segmentation in optical coherence tomography (OCT) images, introducing a new boundary-enhanced encoder-decoder architecture. This architecture aims to precisely extract choroid layer information from images with blurred edges, assisting in choroidal thickness calculations.

## Glaucoma

Glaucoma is a condition that gradually damages the optic nerve, often associated with elevated intraocular pressure. If left uncontrolled, glaucoma may lead to permanent vision loss. Zhang et al. introduce various diagnostic models for glaucoma based on different examined images, for example, random forest models and VGG networks based on visual field test results, ResNet classifier based on fundus images, and ResNet classifier based on OCT. In view of the difficulty of creating standard data sets and standardization guidelines, it is suggested that we should optimize data sets and build multi-center, large sample, and high-efficiency data sets. Finally, the clinical application rules are more standardized, and the diagnosis and prediction tools for glaucoma are simplified in a single direction, benefiting multiple ethnic groups.

## Cataracts

Cataracts involve the gradual clouding of the lens, resulting in decreased vision clarity and difficulty seeing objects. While cataracts can be treated through surgery, untreated cases can severely impair vision. Xie et al. develop a deep learning method for screening visually impaired cataract cases using fundus images. A total of 8,395 fundus images (5,245 subjects) from three clinical centers and corresponding visual function parameters were collected to develop and evaluate the deep learning method for classifying non-cataract, mild cataract, and vision-impaired cataract. They used three deep learning algorithms (DenseNet121, Inception V3, and ResNet50) to train the model to get the best model of the system. The performance of the system is evaluated by the area under the receiver operating characteristic curve (AUC), sensitivity, and specificity. On the internal test dataset as well as two external test datasets, the optimal algorithm was DenseNet121, and the system performed better in detecting visually impaired cataracts than cataract specialists (*p* < 0.05). Research shows that a function-focused screening tool has the potential to identify visually impaired cataracts in fundus images, leading to timely referral of patients to tertiary eye hospitals.

## Retinal diseases

The retina, a thin layer at the back of the eye, plays a vital role in converting light into signals that our brain interprets as vision. Retinal diseases are various medical conditions that affect the retina’s structure or function. Early detection and treatment are crucial for preserving eye health. Bai et al. evaluate the accuracy and reliability of AI approaches using OCT images to achieve community screening for 15 retinal diseases. The OCT scans cover an area of 12 mm × 9 mm at the posterior pole retina involving the macular and optic disc. The 15 retinal diseases include pigment epithelial detachment, posterior vitreous detachment, epiretinal membranes, sub-retinal fluid, choroidal neovascularization, drusen, retinoschisis, cystoid macular edema, exudation, macular hole, retinal detachment, ellipsoid zone disruption, focal choroidal excavation, choroid atrophy, and retinal hemorrhage. Song et al. explore the biological significance of cytokines in the eye using OCT images. They analyze the correlation between cytokine levels in aqueous humor and retinal fluid, shedding light on the potential role of cytokines in the development of neovascular age-related macular degeneration. Moreover, Feng et al. provide comprehensive overviews of the application of AI technologies in macular edema.

## Fundus vasculature disorders

Fundus vascular disorders are a group of medical diseases related to the vascular structure at the base of the eye. Ji et al. discuss the application of AI in the diagnosis and grading of retinal vascular diseases, such as diabetic retinopathy, retinal vein occlusion, and retinopathy of prematurity, based on color fundus photographs. A significant portion of the research in this topic of our Research Topic focuses on examining alterations in microvasculature before and after the onset of disease or following surgical interventions. Shi et al.segment and measure retinal vessels in fundus color photographs, followed by exploring the relationship between vascular parameters, including retinal vessel branching angle, vessel fractal dimension, vessel diameter, vessel tortuosity, vessel density, and cognitive impairment. Deng et al.use OCT angiography (OCTA) images to compare differences in macular microvasculature between type II diabetes patients with and without peripheral neuropathy. Zhang et al. calculate functional parameters related to retinal vessel oxygen saturation based on fundus color photographs to investigate changes in retinal vessel oxygen saturation in type II diabetes patients. Shen et al. use OCTA images to discuss the condition of macular microvasculature and the recovery of visual function in patients after idiopathic epiretinal membrane surgery. Tang et al.discuss changes in retinal microvasculature in patients with moderate to high myopia after implantable collamer lens (ICL) implantation.

In addition to the aforementioned studies on the clinical application of AI, Su et al. propose an attention-guided cascade network for retinal vessel segmentation, aiming to accurately segment retinal vessels from fundus images. Moreover, Zhang et al. conduct research on eliminating eyelash artifacts from ultra-wide-field fundus images, aiming to improve the visibility of retinal vasculature and enhance the quality of eye examination images. These studies enable more precise quantitative analysis of retinal vasculature, which can enhance the accuracy of vascular-related research.

## Conclusion

Research on this topic has covered a wide range of chronic ocular diseases, including ocular surface and orbital diseases, myopia, glaucoma, cataracts, retinal diseases, and vascular diseases, providing us with a comprehensive understanding of chronic ocular conditions. Additionally, the studies within this topic effectively encompass various applications of AI technology in chronic ocular disease research. These applications include the automatic diagnosis of chronic ocular diseases using AI, the exploration of the relationship between changes in ocular structures and diseases or treatments using AI, and the enhancement of medical education and work efficiency using AI. These research efforts have offered valuable insights into the use of AI in the clinical diagnosis, treatment, and management. We are confident that we will continue to make better use of AI technology in the future.

## References

[B1] Anaraki A. K. Ayati M. Kazemi F. (2019). Magnetic resonance imaging-based brain tumor grades classification and grading via convolutional neural networks and genetic algorithms. J]. Biocybern. Biomed. Eng. 39 (1), 63–74. 10.1016/j.bbe.2018.10.004

[B2] Chassagnon G. Vakalopoulou M. Battistella E. Christodoulidis S. Hoang-Thi T. N. Dangeard S. (2021). AI-driven quantification, staging and outcome prediction of COVID-19 pneumonia. Med. image Anal. 67, 101860. 10.1016/j.media.2020.101860 33171345 PMC7558247

[B3] Fang H. Li F. Fu H. Sun X. Cao X. Lin F. (2022). Adam challenge: detecting age-related macular degeneration from fundus images. IEEE Trans. Med. Imaging 41 (10), 2828–2847. 10.1109/TMI.2022.3172773 35507621

[B4] Farshad A. Yeganeh Y. Gehlbach P. (2022). Y-Net: a spatiospectral dual-encoder network for medical image segmentation[C]//International Conference on Medical Image Computing and Computer-Assisted Intervention. Cham: Springer Nature Switzerland, 582–592.

[B5] Fu F. Shan Y. Yang G. Zheng C. Zhang M. Rong D. (2023). Deep learning for head and neck CT angiography: stenosis and plaque classification. Radiology 307 (3), e220996. 10.1148/radiol.220996 36880944

[B6] Fu H. Cheng J. Xu Y. Wong D. W. K. Liu J. Cao X. (2018). Joint optic disc and cup segmentation based on multi-label deep network and polar transformation. IEEE Trans. Med. imaging 37 (7), 1597–1605. 10.1109/TMI.2018.2791488 29969410

[B7] Han D. Liu J. Sun Z. Cui Y. He Y. Yang Z. (2020). Deep learning analysis in coronary computed tomographic angiography imaging for the assessment of patients with coronary artery stenosis. Comput. Methods Programs Biomed. 196, 105651. 10.1016/j.cmpb.2020.105651 32712571

[B8] Hu J. Wang H. Wu G. Cao Z. Mou L. Zhao Y. (2022). Multi-scale interactive network with artery/vein discriminator for retinal vessel classification. IEEE J. Biomed. Health Inf. 26 (8), 3896–3905. 10.1109/JBHI.2022.3165867 35394918

[B9] Li F. Pan J. Yang D. Wu J. Ou Y. Li H. (2022). A multicenter clinical study of the automated fundus screening algorithm. Transl. Vis. Sci. Technol. 11 (7), 22. 10.1167/tvst.11.7.22 PMC9339691 35881410

[B10] Li J. Jin P. Zhu J. Zou H. Xu X. Tang M. (2021). Multi-scale GCN-assisted two-stage network for joint segmentation of retinal layers and discs in peripapillary OCT images. Biomed. Opt. Express 12 (4), 2204–2220. 10.1364/BOE.417212 33996224 PMC8086482

[B11] Morgan R. W. Drance S. M. (1975). Chronic open-angle glaucoma and ocular hypertension. An epidemiological study. Br. J. Ophthalmol. 59 (4), 211–215. 10.1136/bjo.59.4.211 1138846 PMC1042596

[B12] Orlando J. I. Fu H. Breda J. B. van Keer K. Bathula D. R. Diaz-Pinto A. (2020). Refuge challenge: a unified framework for evaluating automated methods for glaucoma assessment from fundus photographs. Med. image Anal. 59, 101570. 10.1016/j.media.2019.101570 31630011

[B14] Schaumberg D. A. Sullivan D. A. Dana M. R. (2002). “Epidemiology of dry eye syndrome,” in Lacrimal Gland, Tear Film, and Dry Eye Syndromes 3: Basic Science and Clinical Relevance Part A and B, 989–998. 10.1007/978-1-4615-0717-8_140 12614022

[B13] Sharafeldin N. Kawaguchi A. Sundaram A. Campbell S. Rudnisky C. Weis E. (2018). Review of economic evaluations of teleophthalmology as a screening strategy for chronic eye disease in adults. Br. J. Ophthalmol. 102 (11), 1485–1491. 10.1136/bjophthalmol-2017-311452 29680803

[B15] Singh H. Singh H. Latief U. Tung G. K. Shahtaghi N. R. Sahajpal N. S. (2022). Myopia, its prevalence, current therapeutic strategy and recent developments: a Review. Indian J. Ophthalmol. 70 (8), 2788–2799. 10.4103/ijo.IJO_2415_21 35918918 PMC9672758

[B16] Wu J. Fang H. Li F. Fu H. Lin F. Li J. (2023). Gamma challenge: glaucoma grading from multi-modality images. Med. Image Anal. 90, 102938. 10.1016/j.media.2023.102938 37806020

[B17] Yang W. Shao Y. Xu Y. Expert Workgroup of Guidelines on Clinical Research Evaluation of Artificial Intelligence in Ophthalmology 2023, Ophthalmic Imaging and Intelligent Medicine Branch of Chinese Medicine Education Association, Intelligent Medicine Committee of Chinese Medicine Education Association (2023). Guidelines on clinical research evaluation of artificial intelligence in ophthalmology (2023). Int. J. Ophthalmol. 16 (9), 1361–1372. 10.18240/ijo.2023.09.02 37724285 PMC10475621

[B18] Zhang X. Xiao Z. Fu H. Hu Y. Yuan J. Xu Y. (2022). Attention to region: region-based integration-and-recalibration networks for nuclear cataract classification using AS-OCT images. Med. Image Anal. 80, 102499. 10.1016/j.media.2022.102499 35704990

